# Pharmacy Customers’ Attitudes Towards Expanded Pharmacy Services in Croatia

**DOI:** 10.3390/pharmacy13010002

**Published:** 2024-12-31

**Authors:** Josipa Bukic, Doris Rusic, Toni Durdov, Kristian Tarabaric, Darko Modun, Dario Leskur, Ana Seselja Perisin, Martin Kondza, Josko Bozic

**Affiliations:** 1 Department of Pharmacy, University of Split School of Medicine, 21000 Split, Croatia; jbukic@mefst.hr (J.B.); toni.durdov@mefst.hr (T.D.); tarabarickristian@gmail.com (K.T.); dmodun@mefst.hr (D.M.); dleskur@mefst.hr (D.L.); aperisin@mefst.hr (A.S.P.); 2 Department of Laboratory Medicine and Pharmacy, Faculty of Medicine, Josip Juraj Strossmayer University of Osijek, 31000 Osijek, Croatia; 3 Faculty of Pharmacy, University of Mostar, 88000 Mostar, Bosnia and Herzegovina; martin.kondza@farf.sum.ba; 4 Faculty of Food and Technology, Josip Juraj Strossmayer University of Osijek, 31000 Osijek, Croatia; 5 Department of Pathophysiology, University of Split School of Medicine, 21000 Split, Croatia; jbozic@mefst.hr

**Keywords:** community pharmacy, new pharmacy services, survey, patient-centered care

## Abstract

Pharmacists have been recognized as the most accessible healthcare professionals, and research has been carried out on expanded pharmacy services they could provide. Additional pharmacy services are a cost-effective way to prevent medication errors, reduce the number of drug-related problems, and prevent chronic disease progression. Therefore, this study aims to evaluate pharmacy service users’ views of expanded pharmacy services in Croatia. This study included 745 participants. Patients who have a healthcare professional in their family more frequently knew of the existence of e-health records and the option to share it with their pharmacists (134, 56.3% vs. 229, 45.2%, *p* = 0.005), while persons that have chronic illness more frequently visit the same pharmacy (176, 77.9% vs. 178, 34.3%, *p* < 0.001). Participants are confident that pharmacists can provide screening services and education on inhaler usage; however, only around 60% agreed that pharmacists can independently lead therapy adjustment, medication substitution, or monitor therapy based on test results. Our findings should be supported with projects evaluating the cost-effectiveness of such services as they would be accepted by a greater number of pharmacy service users if covered by the national health insurer.

## 1. Introduction

Pharmacists are often perceived by patients as the most accessible healthcare professionals because they are available whenever patients walk into the pharmacy [1]. However, for a long period of time, the role of the pharmacist was solely as a medication dispenser so it is unsurprising that pharmacists may still be perceived as vendors by a large proportion of patients [2,3]. Nowadays, the role of the pharmacist is rapidly evolving, with policymakers, business owners, and pharmacists themselves starting to take advantage of pharmacists’ unique position in the healthcare system and this study aims to investigate those opportunities in Croatia [4,5].

Pharmacists are well-positioned to correct prescribing errors and reduce the number of drug-related problems such as drug interactions, contraindications, dosing errors, etc. [4]. Multiple studies have confirmed pharmacists’ capability to reduce drug-related problems and improve patients’ medication adherence, while proper pharmaceutical care could significantly improve patients’ knowledge about chronic diseases [6,7]. Pharmacies are well-equipped with tools necessary for measuring weight, blood pressure, blood sugar, and sometimes even a larger number of diagnostic parameters; therefore, they could play a significant part in identifying risk factors of multiple conditions and recognizing early signs of it [8].

Patients often seek advice about common ailments at the pharmacy, which is usually resolved by the sale of an over-the-counter (OTC) medicine, giving advice without sale, and referring patients to the general practitioner. The pharmacy is refunded for the service only in the first scenario, where the consultation ends with the sale of OTC medicine. A study from New Zealand showed that pharmacists found dealing with minor ailments part of their everyday routine, so it would be beneficial to attract funding for that service to both properly compensate pharmacists for their time and to take some burden off general practitioners’ schedules. The same study showed that participants expressed concern that pharmacies would not be sustainable, only making a profit by dispersing products because of a growing trend of OTC internet sales, making funding additional services necessary. Up to 50% of services pharmacists included in the mentioned study did not end up with reimbursement for given service. More so, the common pharmacy model, which relies solely on the distribution and sales of medicines and other products, does not fully use a pharmacist’s clinical knowledge and capabilities [9].

Additional pharmacy services are a cost-effective way to prevent medication errors, reduce the number of drug-related problems, and prevent chronic disease progression [6]. Recent studies state that patients who received additional pharmacy services related to chronic disease management had improved intermediate clinical outcomes and numbers of adverse drug reactions while also gaining important knowledge and practices regarding their condition, e.g., improving inhaler technique in asthma patients [10,11,12]. Another meta-analysis reported a significant reduction in blood pressure values when an additional service called medication therapy management is implemented by pharmacists and even reducing number of complications from hypertension by almost 9% in ten years. Ultimately, medication therapy management in pharmacies saves costs for the healthcare system and improves patients’ quality of life [13].

There are numerous examples of additional pharmacy services’ successful implementation. The most common service is vaccination in pharmacies, as pharmacists are already involved in vaccination programs across the world. Past experience has shown that the vaccination rate grows in areas with pharmacists performing vaccination alongside medical doctors [14,15]. A great example of additional pharmacy services implementation is the smoking-cessation program in Brazil, while Germany implemented five additional pharmacy services in everyday pharmaceutical care for which pharmacies are reimbursed by insurers [16,17]. Multiple other countries developed additional pharmacy services programs, with the most common services offered being blood pressure measurements, blood sugar measurements, skincare analysis, vaccinations, smoking cessation programs, inhalation checks, and more [18,19,20].

Additional pharmacy services are not recognized in the Croatian healthcare system as they are not paid for by insurers or patients. Although pharmacies are integrated into primary care by law, that is not adequately followed in practice. There was an initiative to include and reimburse additional pharmacy services in Croatian healthcare in 2014 as the Croatian Chamber of Pharmacists published protocols for delivering various services such as blood sugar measurement, blood pressure measurement, inhalation technique check-ups, etc. Efforts to implement expanded services across community pharmacies in Croatia were halted and have not been resumed to this day [21]. There are few postgraduate specialist studies for pharmacists in Croatia, including “Clinical Pharmacy” studies and also specialization in both hospital and public pharmacies where Croatian pharmacists are educated even further to perform additional services but completing those educations means nothing in terms of valuation of services provided by them [22]. Pharmacists often provide said services as part of their everyday work and provide pharmaceutical care to the patients, meaning that, currently, implementation of these services relies only on the personal enthusiasm of pharmacists and is not recognized or reimbursed by the national health insurer [23]. Croatia has an above-average number of pharmacies and pharmacists per capita compared to the European Union average. Croatian pharmacists have to be constantly committed to education to obtain their practicing license, so it would be beneficial and necessary to include pharmacists and their knowledge in the more developed pharmaceutical care concept [22].

Assessing patients’ knowledge, attitudes, and willingness to use and pay for additional pharmacy services is a necessary step for the implementation of expanded pharmacy services nationwide. Since several countries have implemented new medicine services in community pharmacies, we wanted to explore Croatian pharmacy service users’ views on such services and their willingness to fund them out of pocket. A recent Croatian study reported pharmacy users having positive attitudes towards vaccination programs in pharmacies, as they see pharmacists as qualified to perform vaccination. More so, pharmacy users mostly agreed with the vaccination provider being additionally paid for that service [24]. That was the only study conducted in Croatia exploring patients’ attitudes towards additional pharmacy services. Therefore, the aim of this study is to evaluate pharmacy service users’ views of expanded pharmacy services to establish a starting point for the future implementation of said services in the Croatian healthcare system.

## 2. Materials and Methods

All of the participants of this study were adult Croatian residents, regardless of their place of residence, who received an online questionnaire labeled “Questionnaire on Additional Pharmacy Services in the Republic of Croatia” via pseudo snowballing sampling. The assessment of the general population’s attitudes towards the introduction of additional pharmacy services in pharmacies in the Republic of Croatia was conducted through a cross-sectional study using an anonymous questionnaire, which began in April 2024 and lasted for six months.

The survey questionnaire used in the research is presented in the Supplementary Materials. The questionnaire was written in Croatian and was created by the researcher specifically for this study. A literature review was conducted by two pharmacists to assess additional pharmacy services available in European countries and across the world. This list comprised 44 pharmacy services from eight different countries. The list of these services was then forwarded to two community pharmacists employed in the Split-Dalmatia County Pharmacy and educated in specialist studies for pharmacy care in community pharmacy. After consultation with them, 13 additional services that they deemed most likely to be implemented in the near future or most valuable to patients were selected to be included in the final questionnaire. A pilot test was carried out on 10 persons with no healthcare education or background to confirm that the questionnaire was understandable and comprehensive, after which necessary adjustments were made.

The questionnaire is divided into two parts. The first part describes 13 additional pharmacy services that are currently unavailable in the Republic of Croatia. For each additional pharmacy service, there are five statements and, for each statement, participants expressed their level of agreement using a Likert scale ranging from 1 (Strongly Disagree) to 5 (Strongly Agree). The second part of the questionnaire is used to collect sociodemographic data from the respondents, such as sex, age group, average household monthly income, the highest level of education achieved, whether they suffer from chronic illnesses, and more. The questionnaire is presented in the Supplementary Data.

Data were collected via an online Google Forms survey. For the sample size determination, Survey Monkey’s sample size calculator was utilized. Based on the population of Croatia, the calculated sample size was 385 respondents. We used the pseudo snowballing sampling technique. The link was distributed to University of Split School of Medicine students, who then distributed it further to family and friends, meaning the general population. Participation in this study was completely anonymous and voluntary, and the collected data were used for this study, which participants were informed about in the introductory section of the questionnaire. By completing the questionnaire, participants gave their informed consent to take part in this study. This study was approved by the Ethics Committee of the University of Split School of Medicine.

The results are presented as whole numbers and percentages. The chi-square test and Fisher’s exact test, where necessary, were performed to compare answers between groups based on the presence of chronic illness and work status. Statistical analysis was performed using IBM SPSS Statistics software (version 25). Statistical significance was set at *p* < 0.05.

## 3. Results

This study included 745 participants who were dominantly female (76.9%). More than half of the participants were 35 years old or younger, and there were roughly 6% of participants were older than 65. Around 30% reported having a chronic illness. There were 141 students and 560 employed and 44 retired persons surveyed. Roughly one-third reported having a healthcare professional in the family, and little less than half were familiar with the existence of e-health records and an option to share access to these records with their pharmacists (Table 1). Patients who had a healthcare professional in their family more frequently knew of the existence of e-health records and the option to share it with their pharmacists (134, 56.3% vs. 229, 45.2%, *p* = 0.005), while persons that had a chronic illness more frequently visit the same pharmacy (176, 77.9% vs. 178, 34.3%, *p* < 0.001).

Most surveyed persons reported that they understood the described pharmacy service, with a range of 81.6% understanding the described Pharmacist-led therapy adjustment and 97.3% stating that they understood Hypertension screening in the pharmacy, as presented in the Supplementary File Table S1. More than 90% agreed that it would be useful if services on Inhaler usage education, Hypertension screening in pharmacy, and Diabetes screening and education in pharmacy were made available in community pharmacies (Table S2).

Participants’ views on whether questioned expanded pharmacy services can be provided independently by an additionally trained pharmacist are given in Table 2. Participants are confident that pharmacists can provide screening services and education on inhaler usage; only around 60% agreed that pharmacists can independently lead therapy adjustment, medication substitution, or monitor therapy based on test results. Around 15% disagreed with pharmacists providing vaccination services and administering injectable medications.

Most participants agreed that education on how to use inhalers, screening services, and vaccination should be covered by the Croatian Health Insurance Fund, as presented in Table S3. Only around 64% agreed that smoking cessation services should be covered, and around 68% agreed that pharmacist-led therapy adjustment, medication substitution, and access to e-health records for therapy monitoring should be recognized and reimbursed by the national insurer. Table 3 shows that around half of the participants would be willing to pay for the services out of pocket. From these, most would be willing to pay between EUR 6 and 15 per service, as shown in Table S4.

Analysis of the results showed that persons who had a healthcare professional in their family were less likely to agree that listed services can be independently performed by an additionally trained pharmacist for services such as vaccination, medication and vaccination consultations for pregnant women, smoking cessation support, pharmacist-led therapy adjustment, pharmacists administering injectable medications, and entering data into patients’ e-health records (Table 4). Participants in retirement more often agreed that some of the listed services could be performed independently by a trained pharmacist than students and employed persons (Table S5) and that several listed services would be useful if available in pharmacy (Table S6).

## 4. Discussion

The results of this study indicate that pharmacy service users in Croatia are interested in expanded pharmacy services and that they would even be likely to finance such services out of pocket. Around two-thirds of persons who suffer from a chronic illness tend to visit the same pharmacy. This is an outstanding opportunity for pharmacists to track and improve treatment outcomes for these patients through expanded pharmacy services. Studies show that advanced pharmacy services may offer better patient outcomes and reduce healthcare costs [25].

Establishing trust between patients and pharmacists is a prerequisite for providing patient-centered pharmaceutical care. Published studies report that pharmacy service users report trust in pharmacists as moderately high (score 7.4 out of a maximum of 10). Expertise in the pharmacy and personal relationship with the pharmacy were among the elements identified that contribute to building trust [26]. There were also some surprising results in this study. Study participants that reported having a healthcare professional in their family were less likely to agree that additionally trained pharmacist can perform listed services. This finding was significant for vaccination, medication and vaccination consultations for pregnant women, smoking cessation support, pharmacist-led therapy adjustment, pharmacists administering injectable medications, and entering data into patients’ e-health records. As this study did not differentiate from specific healthcare professions when asking about family members, doctors may find some of the expanded services as primarily their scope of work. Pharmacists may experience difficulties differentiating the current pharmaceutical care they provide from the new service [27]. A simple barrier to efficient implementation of new pharmacy services is a lack of awareness among both patients and general practitioners of the implemented services [28]. Other health professionals may deem some of the new services as part of their own tasks and be discouraged from advising and promoting such services to patients [27]. It comes as no surprise that patients who have a healthcare professional in their family more frequently knew of the existence of e-health records and the option to share it with their pharmacists, indicating that some features are not widely recognized by patients and may require increasing awareness among the public.

It is very important to highlight that there were no systematically included pharmacy services in Croatian healthcare. Providing additional services was occasionally utilized by pharmacy owners as a marketing tool. Also, sometimes, various awareness campaigns are conducted with pharmacy services included, such as screening for the most common chronic diseases [29]. Only around 60% of participants agreed that pharmacists could independently lead therapy adjustment, medication substitution, or monitor therapy based on test results, and around 75% agreed that pharmacists could input data into the patient’s e-health record. Agreeing rates were much higher for education on inhaler technique and screening services. Previous studies have demonstrated that pharmacists view the inability to access medical notes and test results as a barrier to patient consultation and setting treatment goals. Moreover, access to clinical data can be a guide to which additional services should be provided to the patient. A barrier to implementation may be general practitioners’ views of community pharmacy services as duplicating their work. A method to overcome this can be training both professions together for services [21,30].

It is not surprising that a greater proportion of participants agreed that pharmacy services should be covered by the national insurer and that between 30 and 50% were willing to finance such services themselves. Willingness to pay out of pocket for expanded pharmacy services may be connected to individuals’ income, as well as the presence of certain illnesses. For example, a study in Australia demonstrated patient’s willingness to pay for asthma management services and their great appreciation for the service [31]. It should be considered that participants in this study included mostly younger adults who may not perceive the total cost of chronic illnesses for an individual and could overestimate their willingness to finance a certain service. This study also demonstrated that retired individuals, who were most likely older, more often agreed that pharmacists could perform listed services independently. This could be linked to their previous experience with pharmacists, as a number of pharmacists offer a number of expanded services but are not compensated for them. Studies found that patients have reported positive experiences with advanced pharmacy services in pharmacies, while pharmacists demonstrated positive attitudes toward providing these services [32].

In this study, education on the use of inhaler devices was rated among the most useful services that may be offered in pharmacy, a service that should be reimbursed by the national insurer, and a service that falls into the scope of pharmacists’ competence. This service is well established, and there is a vast amount of data on improved patient outcomes with asthma management services. For example, in October 2013, community pharmacies in Belgium introduced a New Medicines Service to support new users of inhaler devices. This was the first initiative for advanced pharmaceutical care in community pharmacies in Belgium [27]. Implementation of pharmacy asthma care programs in Australian community pharmacies has resulted in improved asthma control for patients, with patients being 2.7 times more likely to achieve “not severe” status starting from “severe” and increased adherence to medications [33]. A more recent study investigating eHealth intervention Sara Apothecary Respiratory Advice designed to improve inhaler use and provide pharmacists’ follow-up support showed that such intervention may result in a slower increase in exacerbation rates but did not significantly affect patients’ adherence [34]. Implementation of pharmacist-led inhalation technique assessment service in Norwegian pharmacies has resulted in significantly improved inhalation techniques among patients. The majority of patients included in the program expressed a willingness to participate in the program again if prescribed a new inhaler device, and more than half for the same inhaler device [35]. Taking into consideration that most patients with chronic illness tend to visit the same pharmacy and that the Global Initiative for Asthma (GINA) Global Strategy for Asthma Management and Prevention 2024 recommends checking inhaler technique and patient adherence at every opportunity, such service may be a great stepping stone for introduction of expanded pharmacy services at a national level in Croatia [36].

Furthermore, although not covered in this study, patient adherence should not be overlooked. New medicine services targeting patient adherence have proven effective in randomized controlled trials [37]. Economic evaluation of the New Medicine Service that increases patient adherence to treatment has demonstrated increased health and lower overall cost [38].

Screening services for different conditions were also seen as quite beneficial by pharmacy service users in Croatia. Pharmacy screening services for undiagnosed type 2 diabetes have had great success with great satisfaction from both patients and general practitioners [39]. Moreover, the implementation of The Diabetes MedsCheck pharmacy service has had positive impacts on the providers, boosting their engagement with other healthcare professionals, leading to their greater personal satisfaction [40]. This is important, as studies show that a majority of pharmacy service users are willing to make an appointment for a medicine-related service and that pharmacists are likely to underestimate the public’s interest in these services. A positive experience with a new pharmacy service may change a patient’s relationship with the pharmacist and affect the way a pharmacist is perceived as a health professional [41].

In this study, therapy consultations for chronic conditions were better accepted than pharmacist-led therapy adjustment or medication substitution. Previously published studies showed that patient counseling upon introduction of new medicines has proven to positively affect patient adherence and treatment safety [42]. Pharmacists’ counseling can contribute to morbidity reduction as well as a reduction in mortality related to therapy [43].

Around two-thirds of study participants agreed that pharmacists can perform vaccinations. During the COVID-19 pandemic, some countries authorized pharmacists to provide vaccines. Furthermore, pharmacists were the most accessible health workers, and, more often than not, their advice replaced doctors’ advice on medicines due to the unavailability of doctors’ offices at that time, confirming their unique positioning in the healthcare system and their invaluable role in patient care [42].

Around half of the participants in this study would pay for a smoking cessation service. It is well-established that community pharmacies can offer support to people trying to quit smoking [44]. Pharmacists can significantly contribute to smoking cessation, with the literature reporting a from 30 to 50% success rate in pharmacy smoking cessation programs and the cost-effectiveness of such services [45,46,47,48].

It is well established that, upon implementation of new pharmacy services, some challenges may arise, requiring increasing awareness of the new role of pharmacists and increasing communication between pharmacists and other health professionals [49]. These should be addressed before new pharmacy services are implemented at a national level.

This study is not without limitations. A full medication history was not obtained from participants. It is likely that some disease-specific interventions were perceived as more useful to participants who suffer from them. Due to the pseudo snowballing sampling technique, it is likely that most of the study participants are residents of Split-Dalmatia County, which may skew the results of our research. Also, due to the primary distribution coming from students, it is possible that the friends and family they distributed a questionnaire to could have a positive bias towards the topic. Furthermore, the demographics of participants are skewed towards women and younger adults. As this may not appropriately represent the general population in Croatia, it may adequately represent the demographic most likely to use these services in the future. We believe this happened due to the e-distribution of the questionnaire and because, generally, women tend to be more interested in this kind of research. Data are self-reported; hence, it is likely that participants overestimated their willingness to pay for additional pharmacy services out of pocket.

## 5. Conclusions

The results of this study demonstrate that patients with chronic illnesses tend to visit the same pharmacy, which offers a great platform and opportunity for pharmacists to introduce new pharmacy services for these patients. Furthermore, this study offers valuable insight into which pharmacy services would be the most appreciated and accepted among pharmacy service users, such as screening services and education on inhaler technique. Our findings should be supported with projects evaluating the cost-effectiveness of such services as they would be accepted by a greater number of pharmacy service users if covered by the national health insurer.

## Figures and Tables

**Table 1:** Demographic characteristics of participants.

		Count (%)
Sex	Female	573 (76.9)
	Male	172 (23.1)
Age (years)	18–25	211 (28.3)
	26–35	176 (23.6)
	36–45	122 (16.4)
	46–55	123 (16.5)
	56–65	70 (9.4)
	65+	43 (5.8)
Chronic illness present	No	519 (69.8)
	Yes	226 (30.3)
Employment status	Student	141 (18.9)
	Employed	560 (75.2)
	Retired	44 (5.9)
HCP in the family	No	507 (68.1)
	Yes	238 (31.9)
Familiarity with the existence of e-health records and the option to share them with your pharmacists	No	382 (51.3)
	Yes	363 (48.7)

HCP—healthcare professional.

**Table 2:** Count and percentage of participants who agree or strongly agree with the statement “The service can be independently performed by an additionally trained pharmacist”.

	Strongly Disagree and Disagree		Neither Agree nor Disagree		Strongly Agree and Agree	
	Count	%	Count	%	Count	%
Inhaler usage education.	22	3.0%	61	8.2%	662	88.9%
Vaccination of patients in the pharmacy.	111	14.9%	87	11.7%	547	73.4%
Hypertension screening in the pharmacy.	31	4.2%	61	8.2%	653	87.7%
Diabetes screening and education in the pharmacy.	32	4.3%	62	8.3%	651	87.4%
Therapy consultations for chronic conditions.	93	12.5%	88	11.8%	564	75.7%
Medication and vaccination consultations for pregnant women.	70	9.4%	59	7.9%	616	82.7%
Smoking cessation support in the pharmacy.	75	10.1%	85	11.4%	585	78.5%
Pharmacist-led therapy adjustment.	193	25.9%	95	12.8%	457	61.3%
Pharmacist-led medication substitution.	157	21.1%	106	14.2%	482	64.7%
Pharmacist-administered injections.	104	14.0%	84	11.3%	557	74.8%
Pharmacist’s access to patient test results for therapy monitoring.	164	22.0%	106	14.2%	475	63.8%
Pharmacist’s access to patient e-health records for therapy monitoring.	79	10.6%	62	8.3%	604	81.1%
Pharmacist’s ability to input data into the patient’s e-health record.	91	12.2%	89	11.9%	565	75.8%

**Table 3:** Count and percentage of participants who agree or strongly agree with the statement “I am willing to pay for this service”.

	Strongly Disagree and Disagree		Neither Agree nor Disagree		Strongly Agree and Agree	
	Count	%	Count	%	Count	%
Inhaler usage education.	181	24.3%	161	21.6%	403	54.1%
Vaccination of patients in the pharmacy.	244	32.8%	125	16.8%	376	50.5%
Hypertension screening in the pharmacy.	248	33.3%	115	15.4%	382	51.3%
Diabetes screening and education in the pharmacy.	199	26.7%	127	17.0%	419	56.2%
Therapy consultations for chronic conditions.	245	32.9%	130	17.4%	370	49.7%
Medication and vaccination consultations for pregnant women.	231	31.0%	105	14.1%	409	54.9%
Smoking cessation support in the pharmacy.	237	31.8%	130	17.4%	378	50.7%
Pharmacist-led therapy adjustment.	322	43.2%	125	16.8%	298	40.0%
Pharmacist-led medication substitution.	331	44.4%	128	17.2%	286	38.4%
Pharmacist-administered injections.	257	34.5%	124	16.6%	364	48.9%
Pharmacist’s access to patient test results for therapy monitoring.	305	40.9%	138	18.5%	302	40.5%
Pharmacist’s access to patient e-health records for therapy monitoring.	313	42.0%	136	18.3%	296	39.7%
Pharmacist’s ability to input data into the patient’s e-health record.	342	45.9%	126	16.9%	277	37.2%

**Table 4:** Count and percentage of participants who agree or strongly agree with the statement “The service can be independently performed by an additionally trained pharmacist” in reference to having a healthcare professional (HCP) in the family.

	HCP in the Family				
	Yes		No		*p*-Value
	Count	%	Count	%	
Inhaler usage education.	210	88.2%	452	89.2%	0.902
Vaccination of patients in the pharmacy.	156	65.5%	391	77.1%	0.002 *
Hypertension screening in the pharmacy.	206	86.6%	447	88.2%	0.794
Diabetes screening and education in the pharmacy.	201	84.5%	450	88.8%	0.195
Therapy consultations for chronic conditions.	175	73.5%	389	76.7%	0.069
Medication and vaccination consultations for pregnant women.	185	77.7%	431	85.0%	0.045 *
Smoking cessation support in the pharmacy.	173	72.7%	412	81.3%	0.009 *
Pharmacist-led therapy adjustment.	135	56.7%	322	63.5%	0.020 *
Pharmacist-led medication substitution.	148	62.2%	334	65.9%	0.427
Pharmacist-administered injections.	163	68.5%	394	77.7%	0.014 *
Pharmacist’s access to patient test results for therapy monitoring.	140	58.8%	335	66.1%	0.147
Pharmacist’s access to patient e-health records for therapy monitoring.	190	79.8%	414	81.7%	0.637
Pharmacist’s ability to input data into the patient’s e-health record.	173	72.7%	392	77.3%	0.005 *

* *p* < 0.05, Chi-square test.

## Data Availability

Data are available on reasonable request to the corresponding author.

## Supplementary Materials

The following supporting information can be downloaded at: https://www.mdpi.com/article/10.3390/pharmacy13010002/s1, Table S1: Count and percentage of participants who agree or strongly agree with the statement “I understand following pharmacy service”; Table S2: Count and percentage of participants who agree or strongly agree with the statement “It would be useful if the service were available in the pharmacy”; Table S3: Count and percentage of participants who agree or strongly agree with the statement “The service should be covered by the Croatian Health Insurance Fund (HZZO)”; Table S4: Amount Participants Are Willing to Pay for the Service; Table S5: Count and percentage of participants who agree or strongly agree with the statement “The service can be independently performed by an additionally trained pharmacist” with reference to employment status; Table S6: Count and percentage of participants who agree or strongly agree with the statement “It would be helpful if the service were available in the pharmacy” with reference to employment status; Table S7: Count and percentage of participants familiar with the existence of e-health records who agree or strongly agree with the statement “It would be useful if the service were available in the pharmacy”; Table S8: Count and percentage of participants familiar with the existence of e-health records who agree or strongly agree with the statement “The service can be independently performed by an additionally trained pharmacist”; Table S9: Count and percentage of participants familiar with the existence of e-health records who agree or strongly agree with the statement “I am willing to pay for this service”; The Questionnaire (Croatian).
