# Supplementary material for: Pharmacy Customers’ Attitudes Towards Expanded Pharmacy Services in Croatia
Source: Pharmacy (Basel). 2024 Dec 31;13(1):2. doi: 10.3390/pharmacy13010002 (PMC11755540; doi:10.3390/pharmacy13010002)
Supplement: Supplementary file 1 [file pharmacy-13-00002-s001.zip › pharmacy-3367525-supplementary.pdf]

Supplementary data

Table S1. Count and percentage of participants who agree or strongly agree with the statement "I understand following pharmacy service"

	Strongly disagree and Disagree		Neither agree nor disagree		Strongly agree and Agree	
	Count	%	Count	%	Count	%
Inhaler usage education.	18	2.4%	17	2.3%	710	95.3%
Vaccination of patients in the pharmacy.	87	11.7%	45	6.0%	613	82.3%
Hypertension screening in the pharmacy.	9	1.2%	11	1.5%	725	97.3%
Diabetes screening and education in the pharmacy.	12	1.6%	14	1.9%	719	96.5%
Therapy consultations for chronic conditions.	41	5.5%	31	4.2%	673	90.3%
Medication and vaccination consultations for pregnant women.	36	4.8%	10	1.3%	699	93.8%
Smoking cessation support in the pharmacy.	34	4.6%	20	2.7%	691	92.8%
Pharmacist-led therapy adjustment.	107	14.4%	30	4.0%	608	81.6%
Pharmacist-led medication substitution.	94	12.6%	35	4.7%	616	82.7%
Pharmacist-administered injections.	67	9.0%	33	4.4%	645	86.6%
Pharmacist's access to patient test results for therapy monitoring.	96	12.9%	38	5.1%	611	82.0%
Pharmacist's access to patient e-health records for therapy monitoring.	30	4.0%	20	2.7%	695	93.3%

Pharmacist's ability to input data into the patient's e-health record.	41	5.5%	28	3.8%	676	90.7%

Table S2. Count and percentage of participants who agree or strongly agree with the statement "It would be useful if the service were available in the pharmacy"

	Strongly disagree and Disagree		Neither agree nor disagree		Strongly agree and Agree	
	Count	%	Count	%	Count	%
Inhaler usage education.	9	1.2%	40	5.4%	696	93.4%
Vaccination of patients in the pharmacy.	131	17.6%	75	10.1%	539	72.3%
Hypertension screening in the pharmacy.	14	1.9%	55	7.4%	676	90.7%
Diabetes screening and education in the pharmacy.	18	2.4%	46	6.2%	681	91.4%
Therapy consultations for chronic conditions.	81	10.9%	91	12.2%	573	76.9%
Medication and vaccination consultations for pregnant women.	67	9.0%	59	7.9%	619	83.1%
Smoking cessation support in the pharmacy.	85	11.4%	93	12.5%	567	76.1%
Pharmacist-led therapy adjustment.	192	25.8%	101	13.6%	452	60.7%
Pharmacist-led medication substitution.	163	21.9%	108	14.5%	474	63.6%
Pharmacist-administered injections.	109	14.6%	98	13.2%	538	72.2%
Pharmacist's access to patient test results for therapy monitoring.	170	22.8%	104	14.0%	471	63.2%
Pharmacist's access to patient e-health records for therapy monitoring.	68	9.1%	77	10.3%	600	80.5%
Pharmacist's ability to input data into the patient's e-health record.	96	12.9%	83	11.1%	566	76.0%

Table S3. Count and percentage of participants who agree or strongly agree with the statement "The service should be covered by the Croatian Health Insurance Fund (HZZO)"

	Strongly disagree and Disagree		Neither agree nor disagree		Strongly agree and Agree	
	Count	%	Count	%	Count	%
Inhaler usage education.	12	1.6%	44	5.9%	689	92.5%
Vaccination of patients in the pharmacy.	57	7.7%	74	9.9%	614	82.4%
Hypertension screening in the pharmacy.	23	3.1%	62	8.3%	660	88.6%
Diabetes screening and education in the pharmacy.	29	3.9%	62	8.3%	654	87.8%
Therapy consultations for chronic conditions.	66	8.9%	89	11.9%	590	79.2%
Medication and vaccination consultations for pregnant women.	51	6.8%	74	9.9%	620	83.2%
Smoking cessation support in the pharmacy.	154	20.7%	116	15.6%	475	63.8%
Pharmacist-led therapy adjustment.	149	20.0%	90	12.1%	506	67.9%
Pharmacist-led medication substitution.	137	18.4%	95	12.8%	513	68.9%
Pharmacist-administered injections.	90	12.1%	85	11.4%	570	76.5%
Pharmacist's access to patient test results for therapy monitoring.	145	19.5%	95	12.8%	505	67.8%
Pharmacist's access to patient e-health records for therapy monitoring.	79	10.6%	74	9.9%	592	79.5%
Pharmacist's ability to input data into the patient's e-health record.	101	13.6%	100	13.4%	544	73.0%

Table S4. Amount Participants Are Willing to Pay for the Service

	0-5€		6-15€		16-30€		Above 30€	
	Count	%	Count	%	Count	%	Count	%
Inhaler usage education.	142	32.4%	172	39.3%	95	21.7%	29	6.6%
Vaccination of patients in the pharmacy.	96	23.5%	173	42.4%	104	25.5%	35	8.6%
Hypertension screening in the pharmacy.	7	53.8%	6	46.2%	0	0.0%	0	0.0%
Diabetes screening and education in the pharmacy.	154	34.3%	201	44.8%	67	14.9%	27	6.0%
Therapy consultations for chronic conditions.	110	27.8%	172	43.4%	86	21.7%	28	7.1%
Medication and vaccination consultations for pregnant women.	120	27.6%	182	41.9%	84	19.4%	48	11.1%
Smoking cessation support in the pharmacy.	104	25.9%	164	40.8%	91	22.6%	43	10.7%
Pharmacist-led therapy adjustment.	101	31.6%	129	40.3%	52	16.3%	38	11.9%
Pharmacist-led medication substitution.	111	36.0%	130	42.2%	38	12.3%	29	9.4%
Pharmacist-administered injections.	120	31.6%	166	43.7%	62	16.3%	32	8.4%
Pharmacist's access to patient test results for therapy monitoring.	88	27.6%	143	44.8%	66	20.7%	22	6.9%
Pharmacist's access to patient e-health records for therapy monitoring.	114	36.8%	128	41.3%	42	13.5%	26	8.4%

Pharmacist's ability to input data into the patient's e-health record.	108	38.6%	128	45.7%	31	11.1%	13	4.6%

Table S5. Count and percentage of participants who agree or strongly agree with the statement "The service can be independently performed by an additionally trained pharmacist" with reference to employment status

	Employment						P-value
	Students		Employed		Retired		
	Count	%	Count	%	Count	%	
Inhaler usage education.	124	87.9%	498	88.9%	40	90.9%	0.978
Vaccination of patients in the pharmacy.	97	68.8%	412	73.6%	38	86.4%	0.110
Hypertension screening in the pharmacy.	121	85.8%	494	88.2%	38	86.4%	0.166
Diabetes screening and education in the pharmacy.	120	85.1%	492	87.9%	39	88.6%	0.502
Therapy consultations for chronic conditions.	97	68.8%	427	76.3%	40	90.9%	0.041*
Medication and vaccination consultations for pregnant women.	111	78.7%	464	82.9%	41	93.2%	0.064
Smoking cessation support in the pharmacy.	111	78.7%	436	77.9%	38	86.4%	0.077
Pharmacist-led therapy adjustment.	65	46.1%	353	63.0%	39	88.6%	<0.001*
Pharmacist-led medication substitution.	83	58.9%	360	64.3%	39	88.6%	<0.001*
Pharmacist-administered injections.	99	70.2%	420	75.0%	38	86.4%	0.029*
Pharmacist's access to patient test results for therapy monitoring.	79	56.0%	357	63.7%	39	88.6%	<0.001*
Pharmacist's access to patient e-health records for therapy monitoring.	116	82.3%	449	80.2%	39	88.6%	0.343
Pharmacist's ability to input data into the patient's e-health record.	111	78.7%	416	74.3%	38	86.4%	0.112

*p<0.05. Chi-square test ** p<0.05 Fischer's exact test

Table S6. Count and percentage of participants who agree or strongly agree with the statement “It would be helpful if the service were available in the pharmacy” with reference to employment status

	Employment						P-value
	Students		Employed		Retired		
	Count	%	Count	%	Count	%	
Inhaler usage education.	133	94.3%	524	93.6%	39	88.6%	0.309
Vaccination of patients in the pharmacy.	93	66.0%	409	73.0%	37	84.1%	0.029*
Hypertension screening in the pharmacy.	127	90.1%	508	90.7%	41	93.2%	0.032**
Diabetes screening and education in the pharmacy.	130	92.2%	511	91.3%	40	90.9%	0.963
Therapy consultations for chronic conditions.	106	75.2%	428	76.4%	39	88.6%	0.334
Medication and vaccination consultations for pregnant women.	117	83.0%	462	82.5%	40	90.9%	0.089
Smoking cessation support in the pharmacy.	106	75.2%	426	76.1%	35	79.5%	0.550
Pharmacist-led therapy adjustment.	65	46.1%	347	62.0%	40	90.9%	<0.001*
Pharmacist-led medication substitution.	80	56.7%	355	63.4%	39	88.6%	<0.001*
Pharmacist-administered injections.	95	67.4%	408	72.9%	35	79.5%	0.155
Pharmacist's access to patient test results for therapy monitoring.	73	51.8%	363	64.8%	35	79.5%	<0.001*
Pharmacist's access to patient e-health records for therapy monitoring.	119	84.4%	444	79.3%	37	84.1%	0.268
Pharmacist's ability to input data into the patient's e-health record.	109	77.3%	420	75.0%	37	84.1%	0.278

*p<0.05. Chi-square test ** p<0.05 Fischer’s exact test

Table S7. Count and percentage of participants familiar with the existence of e-health records who agree or strongly agree with the statement "It would be useful if the service were available in the pharmacy"

	Awareness of e-health records existence				p-value
	Yes		No		
	Count	%	Count	%	
Inhaler usage education.	345	95.0%	351	93.4%	0.137
Vaccination of patients in the pharmacy.	263	72.5%	276	72.3%	0.780
Hypertension screening in the pharmacy.	331	91.2%	345	90.3%	0.889
Diabetes screening and education in the pharmacy.	339	93.4%	342	89.5%	0.033
Therapy consultations for chronic conditions.	290	79.9%	274	71.7%	0.001*
Medication and vaccination consultations for pregnant women.	311	85.7%	308	80.6%	0.030*
Smoking cessation support in the pharmacy.	281	77.4%	286	74.9%	0.727
Pharmacist-led therapy adjustment.	225	62.0%	227	59.4%	0.135
Pharmacist-led medication substitution.	242	66.7%	232	60.7%	<0.001*
Pharmacist-administered injections.	268	73.8%	270	70.7%	0.006*
Pharmacist's access to patient test results for therapy monitoring.	239	65.8%	232	60.7%	0.075
Pharmacist's access to patient e-health records for therapy monitoring.	305	84.0%	295	77.2%	0.043*
Pharmacist's ability to input data into the patient's e-health record.	273	75.2%	293	76.7%	0.332

Table S8. Count and percentage of participants familiar with the existence of e-health records who agree or strongly agree with the statement “The service can be independently performed by aa additionally trained pharmacist”

	Awareness of e-health records existence				p-value
	Yes		No		
	Count	%	Count	%	
Inhaler usage education.	329	90.6%	333	87.2%	0.288
Vaccination of patients in the pharmacy.	263	72.5%	284	74.3%	0.197
Hypertension screening in the pharmacy.	322	88.7%	331	86.6%	0.609
Diabetes screening and education in the pharmacy.	324	89.3%	327	85.6%	0.329
Therapy consultations for chronic conditions.	290	79.9%	274	71.7%	0.001*
Medication and vaccination consultations for pregnant women.	311	85.7%	305	79.8%	0.013*
Smoking cessation support in the pharmacy.	293	80.7%	292	76.4%	0.311
Pharmacist-led therapy adjustment.	234	64.5%	223	58.4%	0.090
Pharmacist-led medication substitution.	251	69.1%	231	60.5%	<0.001*
Pharmacist-administered injections.	279	76.9%	278	72.8%	0.001*
Pharmacist's access to patient test results for therapy monitoring.	245	67.5%	230	60.2%	0.002*
Pharmacist's access to patient e-health records for therapy monitoring.	304	83.7%	300	78.5%	0.048*
Pharmacist's ability to input data into the patient's e-health record.	280	77.1%	285	74.6%	0.140

Table S9. Count and percentage of participants familiar with the existence of e-health records who agree or strongly agree with the statement "I am willing to pay for this service"

	Awareness of e-health records existence				
	Yes		No		p-value
	Count	%	Count	%	
Inhaler usage education.	212	58.4%	191	50.0%	0.048*
Vaccination of patients in the pharmacy.	191	52.6%	185	48.4%	0.200
Hypertension screening in the pharmacy.	206	56.7%	176	46.1%	0.002*
Diabetes screening and education in the pharmacy.	218	60.1%	201	52.6%	0.122
Therapy consultations for chronic conditions.	197	54.3%	173	45.3%	0.013*
Medication and vaccination consultations for pregnant women.	217	59.8%	192	50.3%	0.031*
Smoking cessation support in the pharmacy.	191	52.6%	187	49.0%	0.484
Pharmacist-led therapy adjustment.	155	42.7%	143	37.4%	0.344
Pharmacist-led medication substitution.	158	43.52%	128	33.5%	0.006*
Pharmacist-administered injections.	192	52.9%	172	45.0%	0.035*
Pharmacist's access to patient test results for therapy monitoring.	161	44.4%	141	36.9%	0.057
Pharmacist's access to patient e-health records for therapy monitoring.	156	43.0%	140	36.6%	0.182
Pharmacist's ability to input data into the patient's e-health record.	145	39.9%	132	34.6%	0.202

The Questionnaire (Croatian)

Upitnik o dodatnim ljekarničkim uslugama u Republici Hrvatskoj

I DIO: PITANJA O DODATNIM LJEKARNIČKIM USLUGAMA

U ovom dijelu upitnika opisano je 13 dodatnih ljekarničkih usluga. Ispod opisa svake usluge nalazi se 5 tvrdnji, a Vi zaokruživanjem broja od 1 do 5 izražavate koliko se slažete s navedenom tvrdnjom pri čemu vrijedi:

1 – u potpunosti se ne slažem
2 - ne slažem se
3 - niti se slažem niti se ne slažem
4 - slažem se
5 - u potpunosti se slažem

1. Pacijentima koji boluju od astme a kojima je liječnik prvi put propisao „pumpicu“ ljekarnik će detaljno objasniti i pokazati kako je pravilno koristiti, čuvati i održavati. Kako bi bio siguran da ga je pacijent razumio ljekarnik će zamoliti pacijenta da pred njim ponovi upute i demonstrira kako pravilno koristiti pumpicu.

a) Razumijem opisanu uslugu

1 2 3 4 5

b) Bilo bi korisno da je usluga dostupna u ljekarni

1 2 3 4 5

c) Uslugu može samostalno izvoditindodatno educirani ljekarnik

1 2 3 4 5

d) Usluga bi trebala biti pokrivena od HZZO-a

1 2 3 4 5

e) Voljan sam platiti ovakvu uslugu

1 2 3 4 5

Ukoliko ste u tvrdnji pod e) zaokružili 4 ili 5 zaokružite iznos koji biste bili spremni platiti za navedenu uslugu:

a) do 5 €
b) 6-15 €
c) 16-30 €
d) više od 30 €

2. Ljekarnici mogu bez potrebne prethodne najave u posebnoj prostoriji unutar ljekarne cijepiti pacijente protiv gripe, HPV virusa, zaušnjaka, tetanusa, hepatitisa A i B, COVIDA-19…

a) Razumijem opisanu uslugu

1 2 3 4 5

b) Bilo bi korisno da je usluga dostupna u ljekarni

1 2 3 4 5

c) Uslugu može samostalno izvoditindodatno educirani ljekarnik
1 2 3 4 5

d) Usluga bi trebala biti pokrivena od HZZO-a
1 2 3 4 5
e) Voljan sam platiti ovakvu uslugu
1 2 3 4 5
Ukoliko ste u tvrdnji pod e) zaokružili 4 ili 5 zaokružite iznos koji biste bili spremni platiti za navedenu uslugu:

a) do 5 €
b) 6-15 €
c) 16-30 €
d) više od 30 €

3. U ljekarnama se provodi obvezno mjerenje arterijskog tlaka svim ljudima iznad 40. godine života s ciljem pronalaska ljudi koji imaju povišen arterijski tlak, a kojima on nije dijagnosticiran. Takve ljude bi se zatim uputilo liječniku na dodatne provjere arterijskog tlaka.
a) Razumijem opisanu uslugu

1 2 3 4 5

b) Bilo bi korisno da je usluga dostupna u ljekarni

1 2 3 4 5

c) Uslugu može samostalno izvoditindodatno educirani ljekarnik
1 2 3 4 5

d) Usluga bi trebala biti pokrivena od HZZO-a
1 2 3 4 5
e) Voljan sam platiti ovakvu uslugu
1 2 3 4 5
Ukoliko ste u tvrdnji pod e) zaokružili 4 ili 5 zaokružite iznos koji biste bili spremni platiti za navedenu uslugu:

a) do 5 €
b) 6-15 €
c) 16-30 €
d) više od 30 €

4. Ljekarnici u ljekarnama vrše mjerenje razine šećera u krvi, a pacijenti ispunjavaju test za procjenu rizika nastanka šećerne bolesti s ciljem otkrivanja ljudi s nedijagnosticiranom šećernom bolesti ili povećanim rizikom za razvoj šećerne bolesti. Ljekarnici također savjetuju pacijente o prevenciji i smanjivanju rizičnih faktora za razvoj šećerne bolesti.
a) Razumijem opisanu uslugu

1 2 3 4 5

b) Bilo bi korisno da je usluga dostupna u ljekarni

1 2 3 4 5

c) Uslugu može samostalno izvoditindodatno educirani ljekarnik
1 2 3 4 5

d) Usluga bi trebala biti pokrivena od HZZO-a
1 2 3 4 5

e) Voljan sam platiti ovakvu uslugu

1 2 3 4 5

Ukoliko ste u tvrdnji pod e) zaokružili 4 ili 5 zaokružite iznos koji biste bili spremni platiti za navedenu uslugu:

a) do 5 €
b) 6-15 €
c) 16-30 €
d) više od 30 €

5. Ljekarnik u posebnoj prostoriji u ljekarni vrši konzultacije s pacijentima koji boluju od neke kronične bolesti, a kojima je u terapiju dodan novi lijek. Pacijenti će na taj način saznati je li novi lijek stvarno potreban u terapiji i koja je njegova svrha u liječenju.

a) Razumijem opisanu uslugu

1 2 3 4 5

b) Bilo bi korisno da je usluga dostupna u ljekarni

1 2 3 4 5

c) Uslugu može samostalno izvoditindodatno educirani ljekarnik

1 2 3 4 5

d) Usluga bi trebala biti pokrivena od HZZO-a

1 2 3 4 5

e) Voljan sam platiti ovakvu uslugu

1 2 3 4 5

Ukoliko ste u tvrdnji pod e) zaokružili 4 ili 5 zaokružite iznos koji biste bili spremni platiti za navedenu uslugu:

a) do 5 €
b) 6-15 €
c) 16-30 €
d) više od 30 €

6. Ljekarnik u posebnoj prostoriji u ljekarni vrši konzultacije s trudnicama o cijepljenju, samoliječenju i primjeni receptnih lijekova tijekom trudnoće. Također odgovara na sva pitanja koja trudnice imaju, a koja su vezana uz sigurnost i primjenu lijekova te dodataka prehrani tijekom trudnoće.

a) Razumijem opisanu uslugu

1 2 3 4 5

b) Bilo bi korisno da je usluga dostupna u ljekarni

1 2 3 4 5

c) Uslugu može samostalno izvoditindodatno educirani ljekarnik

1 2 3 4 5

d) Usluga bi trebala biti pokrivena od HZZO-a

1 2 3 4 5

e) Voljan sam platiti ovakvu uslugu

1 2 3 4 5

Ukoliko ste u tvrdnji pod e) zaokružili 4 ili 5 zaokružite iznos koji biste bili spremni platiti za navedenu uslugu:

a) do 5 €
b) 6-15 €

c) 16-30 €
d) više od 30 €

7. Pacijenti koji su na odvikavanju od pušenja imaju mogućnost u bilo kojoj ljekarni koju odaberu dobiti pomoć u smislu moralne podrške i pružanja potpore te dobivanja lijekova koji će im pomoći u odvikavanju od strane ljekarnika.

a) Razumijem opisanu uslugu

1 2 3 4 5

b) Bilo bi korisno da je usluga dostupna u ljekarni

1 2 3 4 5

c) Uslugu može samostalno izvoditindodatno educirani ljekarnik

1 2 3 4 5

d) Usluga bi trebala biti pokrivena od HZZO-a

1 2 3 4 5

e) Voljan sam platiti ovakvu uslugu

1 2 3 4 5

Ukoliko ste u tvrdnji pod e) zaokružili 4 ili 5 zaokružite iznos koji biste bili spremni platiti za navedenu uslugu:

a) do 5 €
b) 6-15 €
c) 16-30 €
d) više od 30 €

8. Ukoliko ljekarnik smatra da propisana doza, oblik lijeka ili način doziranja nije najprikladniji za pacijenta kojem je lijek propisan ima pravo samostalno promijentiti dozu, oblik lijeka ili način doziranja kako bi liječenje bilo što uspješnije.

a) Razumijem opisanu uslugu

1 2 3 4 5

b) Bilo bi korisno da je usluga dostupna u ljekarni

1 2 3 4 5

c) Uslugu može samostalno izvoditindodatno educirani ljekarnik

1 2 3 4 5

d) Usluga bi trebala biti pokrivena od HZZO-a

1 2 3 4 5

e) Voljan sam platiti ovakvu uslugu

1 2 3 4 5

Ukoliko ste u tvrdnji pod e) zaokružili 4 ili 5 zaokružite iznos koji biste bili spremni platiti za navedenu uslugu:

a) do 5 €
b) 6-15 €
c) 16-30 €
d) više od 30 €

9. Ukoliko ljekarnik zaključi da propisani lijek ne odgovara najbolje pacijentu ima pravo samostalno zamijeniti propisani lijek za neki drugi pod uvjetom da novi lijek pripada istoj terapijskoj skupini kao i stari.

a) Razumijem opisanu uslugu

1 2 3 4 5

b) Bilo bi korisno da je usluga dostupna u ljekarni

1 2 3 4 5

c) Uslugu može samostalno izvoditindodatno educirani ljekarnik
1 2 3 4 5

d) Usluga bi trebala biti pokrivena od HZZO-a
1 2 3 4 5
e) Voljan sam platiti ovakvu uslugu
1 2 3 4 5
Ukoliko ste u tvrdnji pod e) zaokružili 4 ili 5 zaokružite iznos koji biste bili spremni platiti za navedenu uslugu:

a) do 5 €
b) 6-15 €
c) 16-30 €
d) više od 30 €

10. Ljekarnik može pacijentima davati lijek u obliku injekcija (npr. Injekcijska terapija koja se prima jednom mjesečno…).
a) Razumijem opisanu uslugu

1 2 3 4 5

b) Bilo bi korisno da je usluga dostupna u ljekarni

1 2 3 4 5

c) Uslugu može samostalno izvoditindodatno educirani ljekarnik
1 2 3 4 5

d) Usluga bi trebala biti pokrivena od HZZO-a
1 2 3 4 5
e) Voljan sam platiti ovakvu uslugu
1 2 3 4 5
Ukoliko ste u tvrdnji pod e) zaokružili 4 ili 5 zaokružite iznos koji biste bili spremni platiti za navedenu uslugu:

a) do 5 €
b) 6-15 €
c) 16-30 €
d) više od 30 €

11. U svrhu praćenja terapije i koncentracije lijeka, ljekarnik ima pravo naručiti,primiti i interpretirati nalaze svojih pacijenata.
a) Razumijem opisanu uslugu

1 2 3 4 5

b) Bilo bi korisno da je usluga dostupna u ljekarni

1 2 3 4 5

c) Uslugu može samostalno izvoditindodatno educirani ljekarnik
1 2 3 4 5

d) Usluga bi trebala biti pokrivena od HZZO-a
1 2 3 4 5
e) Voljan sam platiti ovakvu uslugu
1 2 3 4 5

Ukoliko ste u tvrdnji pod e) zaokružili 4 ili 5 zaokružite iznos koji biste bili spremni platiti za navedenu uslugu:

a) do 5 €
b) 6-15 €
c) 16-30 €
d) više od 30 €

12. Ljekarnik ima pravo pristupa pacijentovom e-kartonu kako bi imao uvid u sve lijekove koje pacijent prima ili je primao. Na taj način bi se umanjio rizik dupliranja terapije, zlouporabe lijekova, nastanka interakcija među lijekovima…

a) Razumijem opisanu uslugu

1 2 3 4 5

b) Bilo bi korisno da je usluga dostupna u ljekarni

1 2 3 4 5

c) Uslugu može samostalno izvoditindodatno educirani ljekarnik

1 2 3 4 5

d) Usluga bi trebala biti pokrivena od HZZO-a

1 2 3 4 5

e) Voljan sam platiti ovakvu uslugu

1 2 3 4 5

Ukoliko ste u tvrdnji pod e) zaokružili 4 ili 5 zaokružite iznos koji biste bili spremni platiti za navedenu uslugu:

a) do 5 €
b) 6-15 €
c) 16-30 €
d) više od 30 €

13. Ljekarnik ima mogućnost unosa podataka u pacijentov e-karton. (npr. unošenje podataka o izdanim bezreceptnim lijekovima…)

a) Razumijem opisanu uslugu

1 2 3 4 5

b) Bilo bi korisno da je usluga dostupna u ljekarni

1 2 3 4 5

c) Uslugu može samostalno izvoditindodatno educirani ljekarnik

1 2 3 4 5

d) Usluga bi trebala biti pokrivena od HZZO-a

1 2 3 4 5

e) Voljan sam platiti ovakvu uslugu

1 2 3 4 5

Ukoliko ste u tvrdnji pod e) zaokružili 4 ili 5 zaokružite iznos koji biste bili spremni platiti za navedenu uslugu:

a) do 5 €
b) 6-15 €
c) 16-30 €
d) više od 30 €

II DIO: OPĆE INFORMACIJE

Molim Vas da odgovorite na nekoliko pitanja o sebi zaokruživanjem ili nadopisivanjem odgovora:

1. **Spol:** **M** **Ž**
2. **Dobna skupina kojoj pripadate je:**
   a) 18-25
   b) 26-35
   c) 36-45
   d) 46-55
   e) 56-65
   f) 65+

3. **Prosječna mjesečna primanja kućanstva (*u eurima*)**____

4. **Najviši postignuti stupanj obrazovanja:**

a) Osnovno obrazovanje ili manje
b) Trogodišnje strukovno obrazovanje
c) Gimnazijsko srednjoškolsko obrazovanje; četverogodišnje i petogodišnje strukovno srednjoškolsko obrazovanje
d) Sveučilišni preddiplomski studiji; stručni preddiplomski studiji
e) Sveučilišni diplomski studiji; specijalistički diplomski stručni studiji; poslijediplomski specijalistički studiji
f) Poslijediplomski znanstveni magistarski studiji ili poslijediplomski sveučilišni (doktorski) studiji

5. Bolujete li od kronične bolesti? **(*ukoliko DA navedite koje*)**

DA **NE** ____

6. Zanimanje kojim se bavite: ____

7. Je li netko u Vašoj obitelji zdravstveni radnik?

DA **NE**

8. Prilikom odlaska u ljekarnu imate li naviku posjećivati uvijek istu ljekarnu ?
(*ukoliko DA navedite ime ljekarne; npr.Ljekarna Brda, Ljekarna Lučac…)*

DA **NE** ____

9. Jeste li upoznati da u sustavu portal.zdravlje.hr (e-građani) postoji Vaš e-karton te da klikom na opciju možete svome ljekarniku dati pristup Vašem kartonu?

DA

NE

Hvala Vam na sudjelovanju!
